# Tris(*N*-benzyl-*N*-methyl­dithio­carbamato-κ^2^ *S*,*S*′)(1,10-phenanthroline-κ^2^ *N*,*N*′)europium(III)

**DOI:** 10.1107/S160053680904135X

**Published:** 2009-10-17

**Authors:** Ibrahim Baba, Indah Raya, Bohari M. Yamin, Seik Weng Ng

**Affiliations:** a School of Chemical Sciences, Universiti Kebangsaan Malaysia, 43600 Bangi, Selangor Darul Ehsan, Malaysia; b Department of Chemistry, University of Malaya, 50603 Kuala Lumpur, Malaysia

## Abstract

In the title compound, [Eu(C_9_H_10_NS_2_)_3_(C_12_H_8_N_2_)], the Eu^III^ atom exists in a distorted square-anti­prismatic coordination geometry. Both dithio­carbamate and the *N*-heterocyclic ligands function in a chelating mode.

## Related literature

For the crystal structures of other europium dithio­carbamate–1,10-phenanthroline adducts see: Regulacio *et al.* (2005 ▶); Su *et al.* (1996 ▶); Varand *et al.* (1996 ▶).
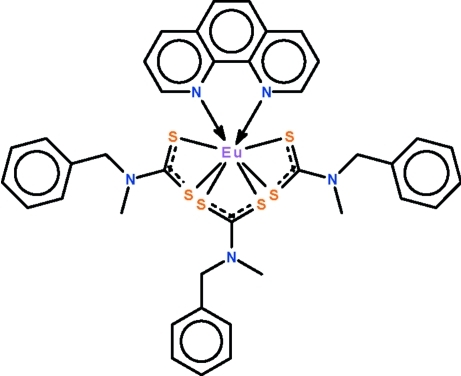


## Experimental

### 

#### Crystal data

[Eu(C_9_H_10_NS_2_)_3_(C_12_H_8_N_2_)]
*M* *_r_* = 921.06
Triclinic, 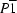

*a* = 10.691 (1) Å
*b* = 12.288 (1) Å
*c* = 16.553 (2) Å
α = 73.652 (2)°
β = 74.720 (2)°
γ = 71.629 (2)°
*V* = 1943.5 (3) Å^3^
*Z* = 2
Mo *K*α radiation
μ = 1.97 mm^−1^
*T* = 293 K
0.48 × 0.35 × 0.20 mm

#### Data collection

Bruker SMART APEX diffractometer
Absorption correction: multi-scan (*SADABS*; Sheldrick, 1996 ▶) *T*_min_ = 0.451, *T*_max_ = 0.694
21775 measured reflections
8695 independent reflections
7425 reflections with *I* > 2σ(*I*)
*R*_int_ = 0.040

#### Refinement

*R*[*F* ^2^ > 2σ(*F* ^2^)] = 0.051
*wR*(*F* ^2^) = 0.141
*S* = 1.08
8695 reflections
463 parameters
H-atom parameters constrained
Δρ_max_ = 3.56 e Å^−3^
Δρ_min_ = −1.65 e Å^−3^

### 

Data collection: *SMART* (Bruker, 2000 ▶); cell refinement: *SAINT* (Bruker, 2000 ▶); data reduction: *SAINT*; program(s) used to solve structure: *SHELXS97* (Sheldrick, 2008 ▶); program(s) used to refine structure: *SHELXL97* (Sheldrick, 2008 ▶); molecular graphics: *X-SEED* (Barbour, 2001 ▶); software used to prepare material for publication: *publCIF* (Westrip, 2009 ▶).

## Supplementary Material

Crystal structure: contains datablocks global, I. DOI: 10.1107/S160053680904135X/xu2634sup1.cif

Structure factors: contains datablocks I. DOI: 10.1107/S160053680904135X/xu2634Isup2.hkl

Additional supplementary materials:  crystallographic information; 3D view; checkCIF report

## Figures and Tables

**Table 1:** Selected bond lengths (Å)

Eu1—N5	2.540 (4)
Eu1—N4	2.569 (4)
Eu1—S4	2.8451 (13)
Eu1—S3	2.8523 (14)
Eu1—S2	2.8627 (13)
Eu1—S5	2.8781 (12)
Eu1—S6	2.8859 (12)
Eu1—S1	2.8970 (12)

**Fig. 1.**
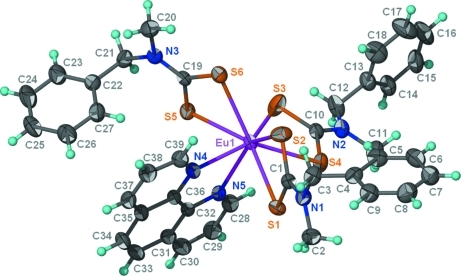
Thermal ellipsoid plot (Barbour, 2001) of Eu(C12H8N2)(C9H8NS2)3 at the 50% probability level. Hydrogen atoms are drawn as spheres of arbitrary radius.

**Fig. 2.**
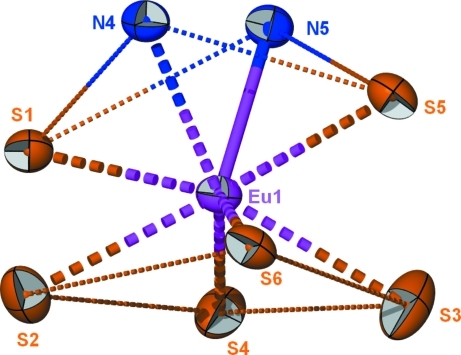
Eight-coordinate coordination geometry of Eu.

## supplementary crystallographic information

### Experimental

Eeuropium(III) chloride (10 mmol) was reacted with 1,10-phenanthroline (10 mmol) in boiling water (15 ml). The solution was then cooled to 280 K. Separately, benzylmethylamine (30 mmol), carbon disulfide (30 mmol) and potassium hydroxide (30 mmol) were reacted in ethanol (15 ml) at 280 K. The two solutions were mixed. The white solid that precipitated was collected and recrystallized from ethanol.

### Refinement

Carbon-bound H-atoms were placed in calculated positions (C—H 0.93 to 0.97 Å) and were included in the refinement in the riding model approximation, with *U*(H) set to 1.2 to 1.5*U*(C). The final difference Fourier map has a large peak/deep hole in the vicinity of Eu1.

### Crystal data

**Table:**

[Eu(C_9_H_10_NS_2_)_3_(C_12_H_8_N_2_)]	*Z* = 2
*M**_r_* = 921.06	*F*(000) = 932
Triclinic, *P*1	*D*_x_ = 1.574 Mg m^−^^3^
Hall symbol: -P 1	Mo *K*α radiation, λ = 0.71073 Å
*a* = 10.691 (1) Å	Cell parameters from 946 reflections
*b* = 12.288 (1) Å	θ = 2.3–28.1°
*c* = 16.553 (2) Å	µ = 1.97 mm^−^^1^
α = 73.652 (2)°	*T* = 293 K
β = 74.720 (2)°	Block, brown
γ = 71.629 (2)°	0.48 × 0.35 × 0.20 mm
*V* = 1943.5 (3) Å^3^	

### Data collection

**Table:**

Bruker SMART APEX diffractometer	8695 independent reflections
Radiation source: fine-focus sealed tube	7425 reflections with *I* > 2σ(*I*)
graphite	*R*_int_ = 0.040
Detector resolution: 8.33 pixels mm^-1^	θ_max_ = 27.5°, θ_min_ = 1.3°
φ and ω scans	*h* = −13→13
Absorption correction: multi-scan (*SADABS*; Sheldrick, 1996)	*k* = −15→15
*T*_min_ = 0.451, *T*_max_ = 0.694	*l* = −21→21
21775 measured reflections	

### Refinement

**Table:**

Refinement on *F*^2^	Primary atom site location: structure-invariant direct methods
Least-squares matrix: full	Secondary atom site location: difference Fourier map
*R*[*F*^2^ > 2σ(*F*^2^)] = 0.051	Hydrogen site location: inferred from neighbouring sites
*wR*(*F*^2^) = 0.141	H-atom parameters constrained
*S* = 1.08	*w* = 1/[σ^2^(*F*_o_^2^) + (0.0982*P*)^2^] where *P* = (*F*_o_^2^ + 2*F*_c_^2^)/3
8695 reflections	(Δ/σ)_max_ = 0.001
463 parameters	Δρ_max_ = 3.56 e Å^−^^3^
0 restraints	Δρ_min_ = −1.65 e Å^−^^3^

### Special details

**Table:**

Geometry. All e.s.d.'s (except the e.s.d. in the dihedral angle between two l.s. planes) are estimated using the full covariance matrix. The cell e.s.d.'s are taken into account individually in the estimation of e.s.d.'s in distances, angles and torsion angles; correlations between e.s.d.'s in cell parameters are only used when they are defined by crystal symmetry. An approximate (isotropic) treatment of cell e.s.d.'s is used for estimating e.s.d.'s involving l.s. planes.

### Fractional atomic coordinates and isotropic or equivalent isotropic displacement parameters (Å^2^)

**Table:**

	*x*	*y*	*z*	*U*_iso_*/*U*_eq_	
Eu1	0.74676 (2)	0.689048 (17)	0.257329 (13)	0.03679 (10)	
S1	0.66925 (12)	0.77165 (10)	0.09104 (7)	0.0440 (3)	
S2	0.81829 (16)	0.89432 (12)	0.14613 (9)	0.0563 (3)	
S3	0.58827 (15)	0.68170 (17)	0.42600 (9)	0.0732 (5)	
S4	0.46969 (14)	0.81089 (12)	0.27570 (8)	0.0568 (3)	
S5	0.88318 (12)	0.48647 (10)	0.36901 (8)	0.0437 (2)	
S6	0.97198 (13)	0.70423 (10)	0.31481 (8)	0.0460 (3)	
N1	0.7477 (4)	0.9584 (3)	−0.0077 (2)	0.0444 (8)	
N2	0.3278 (4)	0.7922 (4)	0.4342 (3)	0.0479 (9)	
N3	1.1131 (4)	0.5000 (3)	0.3939 (2)	0.0439 (8)	
N4	0.9334 (4)	0.5677 (3)	0.1596 (2)	0.0393 (8)	
N5	0.6988 (4)	0.5086 (3)	0.2389 (2)	0.0389 (8)	
C1	0.7458 (4)	0.8818 (4)	0.0689 (3)	0.0407 (9)	
C2	0.6808 (6)	0.9534 (5)	−0.0728 (3)	0.0577 (13)	
H2A	0.6126	0.9121	−0.0458	0.087*	
H2B	0.7455	0.9132	−0.1143	0.087*	
H2C	0.6403	1.0318	−0.1008	0.087*	
C3	0.8156 (5)	1.0535 (4)	−0.0322 (3)	0.0526 (12)	
H3A	0.8786	1.0477	−0.0860	0.063*	
H3B	0.8660	1.0445	0.0112	0.063*	
C4	0.7169 (5)	1.1726 (4)	−0.0420 (3)	0.0465 (10)	
C5	0.6238 (6)	1.2077 (5)	0.0273 (3)	0.0554 (12)	
H5	0.6231	1.1579	0.0813	0.066*	
C6	0.5311 (6)	1.3166 (5)	0.0173 (4)	0.0627 (14)	
H6	0.4688	1.3392	0.0646	0.075*	
C7	0.5309 (6)	1.3905 (5)	−0.0613 (5)	0.0666 (15)	
H7	0.4679	1.4631	−0.0680	0.080*	
C8	0.6242 (7)	1.3573 (5)	−0.1307 (4)	0.0667 (15)	
H8	0.6256	1.4082	−0.1842	0.080*	
C9	0.7156 (6)	1.2490 (5)	−0.1215 (3)	0.0551 (12)	
H9	0.7772	1.2269	−0.1692	0.066*	
C10	0.4478 (5)	0.7666 (4)	0.3853 (3)	0.0414 (9)	
C11	0.2099 (6)	0.8641 (6)	0.3984 (4)	0.0676 (16)	
H11A	0.2361	0.9200	0.3479	0.101*	
H11B	0.1466	0.9051	0.4400	0.101*	
H11C	0.1694	0.8148	0.3836	0.101*	
C12	0.3024 (6)	0.7410 (4)	0.5268 (3)	0.0590 (14)	
H12A	0.3876	0.6988	0.5443	0.071*	
H12B	0.2505	0.6846	0.5374	0.071*	
C13	0.2287 (5)	0.8296 (4)	0.5812 (3)	0.0501 (11)	
C14	0.1026 (6)	0.8280 (5)	0.6291 (3)	0.0581 (13)	
H14	0.0611	0.7740	0.6254	0.070*	
C15	0.0368 (7)	0.9051 (5)	0.6825 (4)	0.0694 (16)	
H15	−0.0477	0.9021	0.7154	0.083*	
C16	0.0970 (9)	0.9863 (6)	0.6868 (4)	0.086 (2)	
H16	0.0530	1.0383	0.7229	0.103*	
C17	0.2201 (10)	0.9912 (7)	0.6387 (5)	0.091 (2)	
H17	0.2589	1.0481	0.6407	0.110*	
C18	0.2885 (7)	0.9115 (6)	0.5866 (4)	0.0727 (17)	
H18	0.3743	0.9131	0.5554	0.087*	
C19	1.0011 (4)	0.5575 (4)	0.3627 (3)	0.0364 (8)	
C20	1.2179 (5)	0.5553 (5)	0.3872 (4)	0.0560 (12)	
H20A	1.2487	0.5880	0.3279	0.084*	
H20B	1.2915	0.4979	0.4104	0.084*	
H20C	1.1830	0.6168	0.4186	0.084*	
C21	1.1386 (5)	0.3742 (4)	0.4357 (3)	0.0471 (11)	
H21A	1.0532	0.3546	0.4591	0.057*	
H21B	1.1829	0.3602	0.4832	0.057*	
C22	1.2240 (5)	0.2934 (4)	0.3764 (3)	0.0437 (10)	
C23	1.3054 (7)	0.1868 (5)	0.4098 (4)	0.0637 (15)	
H23	1.3098	0.1671	0.4676	0.076*	
C24	1.3804 (8)	0.1091 (5)	0.3577 (5)	0.0799 (19)	
H24	1.4345	0.0375	0.3811	0.096*	
C25	1.3762 (7)	0.1359 (5)	0.2727 (4)	0.0694 (16)	
H25	1.4265	0.0832	0.2381	0.083*	
C26	1.2970 (6)	0.2412 (6)	0.2395 (4)	0.0661 (15)	
H26	1.2938	0.2605	0.1814	0.079*	
C27	1.2210 (5)	0.3205 (5)	0.2903 (3)	0.0553 (12)	
H27	1.1678	0.3922	0.2662	0.066*	
C28	0.5873 (5)	0.4759 (4)	0.2804 (3)	0.0488 (11)	
H28	0.5237	0.5229	0.3155	0.059*	
C29	0.5608 (5)	0.3747 (5)	0.2739 (4)	0.0550 (12)	
H29	0.4805	0.3563	0.3031	0.066*	
C30	0.6523 (6)	0.3040 (4)	0.2252 (3)	0.0540 (12)	
H30	0.6361	0.2358	0.2211	0.065*	
C31	0.7737 (5)	0.3339 (4)	0.1800 (3)	0.0438 (10)	
C32	0.7923 (4)	0.4368 (4)	0.1904 (3)	0.0376 (9)	
C33	0.8754 (6)	0.2628 (4)	0.1285 (3)	0.0550 (13)	
H33	0.8613	0.1956	0.1211	0.066*	
C34	0.9919 (6)	0.2908 (4)	0.0902 (3)	0.0537 (12)	
H34	1.0576	0.2424	0.0571	0.064*	
C35	1.0163 (5)	0.3941 (4)	0.0998 (3)	0.0443 (10)	
C36	0.9166 (4)	0.4682 (4)	0.1487 (3)	0.0372 (9)	
C37	1.1370 (5)	0.4265 (5)	0.0607 (3)	0.0520 (12)	
H37	1.2053	0.3799	0.0276	0.062*	
C38	1.1533 (5)	0.5270 (5)	0.0718 (3)	0.0529 (12)	
H38	1.2324	0.5498	0.0464	0.063*	
C39	1.0476 (5)	0.5953 (5)	0.1225 (3)	0.0459 (10)	
H39	1.0591	0.6634	0.1302	0.055*	

### Atomic displacement parameters (Å^2^)

**Table:**

	*U*^11^	*U*^22^	*U*^33^	*U*^12^	*U*^13^	*U*^23^
Eu1	0.03964 (14)	0.02894 (13)	0.04105 (14)	−0.00537 (9)	−0.00616 (9)	−0.01192 (9)
S1	0.0525 (6)	0.0400 (6)	0.0444 (6)	−0.0150 (5)	−0.0092 (5)	−0.0136 (4)
S2	0.0776 (9)	0.0503 (7)	0.0541 (7)	−0.0315 (7)	−0.0285 (6)	−0.0003 (5)
S3	0.0493 (8)	0.1024 (13)	0.0442 (7)	0.0127 (7)	−0.0121 (6)	−0.0132 (7)
S4	0.0528 (7)	0.0582 (8)	0.0435 (6)	0.0107 (6)	−0.0115 (5)	−0.0126 (5)
S5	0.0435 (6)	0.0329 (5)	0.0548 (6)	−0.0110 (4)	−0.0120 (5)	−0.0060 (4)
S6	0.0527 (7)	0.0319 (5)	0.0566 (7)	−0.0130 (5)	−0.0139 (5)	−0.0089 (5)
N1	0.049 (2)	0.0374 (19)	0.044 (2)	−0.0091 (16)	−0.0110 (17)	−0.0051 (15)
N2	0.041 (2)	0.045 (2)	0.051 (2)	−0.0045 (17)	−0.0038 (17)	−0.0131 (17)
N3	0.045 (2)	0.042 (2)	0.047 (2)	−0.0088 (16)	−0.0111 (16)	−0.0130 (16)
N4	0.0377 (19)	0.0356 (18)	0.0443 (19)	−0.0076 (15)	−0.0053 (15)	−0.0126 (15)
N5	0.0360 (18)	0.0360 (18)	0.0449 (19)	−0.0065 (14)	−0.0098 (15)	−0.0105 (15)
C1	0.043 (2)	0.037 (2)	0.042 (2)	−0.0056 (18)	−0.0112 (18)	−0.0116 (17)
C2	0.073 (4)	0.058 (3)	0.046 (3)	−0.012 (3)	−0.022 (2)	−0.012 (2)
C3	0.047 (3)	0.049 (3)	0.055 (3)	−0.015 (2)	−0.010 (2)	0.001 (2)
C4	0.048 (3)	0.044 (2)	0.051 (3)	−0.020 (2)	−0.014 (2)	−0.002 (2)
C5	0.064 (3)	0.056 (3)	0.051 (3)	−0.025 (3)	−0.009 (2)	−0.009 (2)
C6	0.061 (3)	0.062 (3)	0.073 (4)	−0.019 (3)	−0.005 (3)	−0.030 (3)
C7	0.069 (4)	0.043 (3)	0.097 (5)	−0.012 (3)	−0.031 (3)	−0.018 (3)
C8	0.082 (4)	0.047 (3)	0.073 (4)	−0.019 (3)	−0.032 (3)	0.002 (3)
C9	0.063 (3)	0.050 (3)	0.050 (3)	−0.017 (2)	−0.012 (2)	−0.005 (2)
C10	0.043 (2)	0.038 (2)	0.041 (2)	−0.0041 (18)	−0.0064 (18)	−0.0127 (17)
C11	0.047 (3)	0.071 (4)	0.080 (4)	0.001 (3)	−0.018 (3)	−0.022 (3)
C12	0.068 (3)	0.038 (3)	0.058 (3)	−0.009 (2)	0.006 (3)	−0.011 (2)
C13	0.062 (3)	0.035 (2)	0.047 (2)	−0.009 (2)	−0.006 (2)	−0.0068 (19)
C14	0.059 (3)	0.049 (3)	0.057 (3)	−0.003 (2)	−0.003 (2)	−0.016 (2)
C15	0.066 (4)	0.056 (3)	0.063 (3)	0.010 (3)	−0.005 (3)	−0.015 (3)
C16	0.136 (7)	0.045 (3)	0.065 (4)	0.001 (4)	−0.020 (4)	−0.021 (3)
C17	0.140 (7)	0.064 (4)	0.087 (5)	−0.042 (5)	−0.022 (5)	−0.027 (4)
C18	0.083 (4)	0.065 (4)	0.071 (4)	−0.032 (3)	−0.005 (3)	−0.011 (3)
C19	0.040 (2)	0.034 (2)	0.0358 (19)	−0.0082 (17)	−0.0032 (16)	−0.0135 (16)
C20	0.050 (3)	0.061 (3)	0.064 (3)	−0.017 (2)	−0.015 (2)	−0.017 (3)
C21	0.045 (2)	0.048 (3)	0.042 (2)	−0.004 (2)	−0.0115 (19)	−0.0051 (19)
C22	0.045 (2)	0.039 (2)	0.050 (2)	−0.0120 (19)	−0.011 (2)	−0.0097 (19)
C23	0.087 (4)	0.040 (3)	0.059 (3)	−0.004 (3)	−0.023 (3)	−0.007 (2)
C24	0.105 (5)	0.036 (3)	0.092 (5)	0.003 (3)	−0.031 (4)	−0.017 (3)
C25	0.073 (4)	0.051 (3)	0.085 (4)	−0.011 (3)	−0.005 (3)	−0.033 (3)
C26	0.073 (4)	0.065 (4)	0.058 (3)	−0.010 (3)	−0.008 (3)	−0.023 (3)
C27	0.053 (3)	0.052 (3)	0.052 (3)	−0.001 (2)	−0.009 (2)	−0.013 (2)
C28	0.043 (2)	0.047 (3)	0.058 (3)	−0.013 (2)	−0.007 (2)	−0.013 (2)
C29	0.050 (3)	0.049 (3)	0.069 (3)	−0.019 (2)	−0.014 (2)	−0.009 (2)
C30	0.068 (3)	0.038 (2)	0.065 (3)	−0.023 (2)	−0.020 (3)	−0.008 (2)
C31	0.056 (3)	0.032 (2)	0.047 (2)	−0.0099 (19)	−0.020 (2)	−0.0066 (18)
C32	0.042 (2)	0.0299 (19)	0.044 (2)	−0.0044 (16)	−0.0164 (18)	−0.0099 (16)
C33	0.077 (4)	0.037 (2)	0.057 (3)	−0.007 (2)	−0.024 (3)	−0.018 (2)
C34	0.064 (3)	0.041 (3)	0.053 (3)	0.002 (2)	−0.013 (2)	−0.022 (2)
C35	0.047 (2)	0.041 (2)	0.039 (2)	0.0018 (19)	−0.0105 (19)	−0.0123 (18)
C36	0.040 (2)	0.034 (2)	0.037 (2)	−0.0033 (17)	−0.0100 (17)	−0.0114 (16)
C37	0.042 (3)	0.061 (3)	0.048 (3)	0.003 (2)	−0.007 (2)	−0.024 (2)
C38	0.041 (2)	0.066 (3)	0.049 (3)	−0.013 (2)	0.000 (2)	−0.017 (2)
C39	0.039 (2)	0.052 (3)	0.047 (2)	−0.013 (2)	−0.0019 (19)	−0.016 (2)

### Geometric parameters (Å, °)

**Table:**

Eu1—N5	2.540 (4)	C12—H12B	0.9700
Eu1—N4	2.569 (4)	C13—C14	1.375 (7)
Eu1—S4	2.8451 (13)	C13—C18	1.384 (8)
Eu1—S3	2.8523 (14)	C14—C15	1.377 (8)
Eu1—S2	2.8627 (13)	C14—H14	0.9300
Eu1—S5	2.8781 (12)	C15—C16	1.372 (10)
Eu1—S6	2.8859 (12)	C15—H15	0.9300
Eu1—S1	2.8970 (12)	C16—C17	1.357 (12)
S1—C1	1.702 (5)	C16—H16	0.9300
S2—C1	1.723 (5)	C17—C18	1.389 (10)
S3—C10	1.717 (5)	C17—H17	0.9300
S4—C10	1.716 (5)	C18—H18	0.9300
S5—C19	1.713 (4)	C20—H20A	0.9600
S6—C19	1.717 (4)	C20—H20B	0.9600
N1—C1	1.349 (6)	C20—H20C	0.9600
N1—C2	1.465 (6)	C21—C22	1.512 (7)
N1—C3	1.473 (6)	C21—H21A	0.9700
N2—C10	1.320 (6)	C21—H21B	0.9700
N2—C11	1.455 (7)	C22—C27	1.376 (7)
N2—C12	1.471 (7)	C22—C23	1.380 (7)
N3—C19	1.337 (6)	C23—C24	1.384 (9)
N3—C20	1.450 (6)	C23—H23	0.9300
N3—C21	1.475 (6)	C24—C25	1.360 (10)
N4—C39	1.315 (6)	C24—H24	0.9300
N4—C36	1.357 (5)	C25—C26	1.360 (9)
N5—C28	1.330 (6)	C25—H25	0.9300
N5—C32	1.357 (5)	C26—C27	1.385 (8)
C2—H2A	0.9600	C26—H26	0.9300
C2—H2B	0.9600	C27—H27	0.9300
C2—H2C	0.9600	C28—C29	1.397 (7)
C3—C4	1.505 (7)	C28—H28	0.9300
C3—H3A	0.9700	C29—C30	1.344 (8)
C3—H3B	0.9700	C29—H29	0.9300
C4—C5	1.377 (7)	C30—C31	1.419 (7)
C4—C9	1.386 (7)	C30—H30	0.9300
C5—C6	1.388 (8)	C31—C32	1.402 (6)
C5—H5	0.9300	C31—C33	1.415 (7)
C6—C7	1.361 (9)	C32—C36	1.443 (6)
C6—H6	0.9300	C33—C34	1.343 (8)
C7—C8	1.372 (9)	C33—H33	0.9300
C7—H7	0.9300	C34—C35	1.431 (7)
C8—C9	1.376 (8)	C34—H34	0.9300
C8—H8	0.9300	C35—C36	1.403 (6)
C9—H9	0.9300	C35—C37	1.408 (7)
C11—H11A	0.9600	C37—C38	1.367 (8)
C11—H11B	0.9600	C37—H37	0.9300
C11—H11C	0.9600	C38—C39	1.410 (7)
C12—C13	1.501 (7)	C38—H38	0.9300
C12—H12A	0.9700	C39—H39	0.9300
			
N5—Eu1—N4	64.41 (11)	N2—C12—H12A	108.7
N5—Eu1—S4	91.49 (9)	C13—C12—H12A	108.7
N4—Eu1—S4	141.09 (9)	N2—C12—H12B	108.7
N5—Eu1—S3	94.45 (9)	C13—C12—H12B	108.7
N4—Eu1—S3	144.46 (9)	H12A—C12—H12B	107.6
S4—Eu1—S3	61.51 (4)	C14—C13—C18	118.7 (5)
N5—Eu1—S2	136.16 (9)	C14—C13—C12	120.6 (5)
N4—Eu1—S2	87.66 (9)	C18—C13—C12	120.6 (5)
S4—Eu1—S2	90.73 (4)	C13—C14—C15	121.1 (6)
S3—Eu1—S2	124.44 (5)	C13—C14—H14	119.4
N5—Eu1—S5	71.80 (8)	C15—C14—H14	119.4
N4—Eu1—S5	73.60 (9)	C16—C15—C14	119.4 (6)
S4—Eu1—S5	129.73 (4)	C16—C15—H15	120.3
S3—Eu1—S5	72.65 (4)	C14—C15—H15	120.3
S2—Eu1—S5	134.15 (4)	C17—C16—C15	120.5 (6)
N5—Eu1—S6	128.70 (8)	C17—C16—H16	119.7
N4—Eu1—S6	82.53 (9)	C15—C16—H16	119.7
S4—Eu1—S6	134.30 (4)	C16—C17—C18	120.2 (7)
S3—Eu1—S6	90.97 (4)	C16—C17—H17	119.9
S2—Eu1—S6	75.11 (4)	C18—C17—H17	119.9
S5—Eu1—S6	61.43 (3)	C13—C18—C17	119.9 (7)
N5—Eu1—S1	78.24 (8)	C13—C18—H18	120.0
N4—Eu1—S1	75.12 (9)	C17—C18—H18	120.0
S4—Eu1—S1	70.08 (4)	N3—C19—S5	120.9 (3)
S3—Eu1—S1	130.85 (4)	N3—C19—S6	120.9 (3)
S2—Eu1—S1	61.59 (3)	S5—C19—S6	118.2 (3)
S5—Eu1—S1	143.61 (3)	N3—C20—H20A	109.5
S6—Eu1—S1	131.41 (3)	N3—C20—H20B	109.5
C1—S1—Eu1	89.39 (15)	H20A—C20—H20B	109.5
C1—S2—Eu1	90.12 (16)	N3—C20—H20C	109.5
C10—S3—Eu1	91.03 (16)	H20A—C20—H20C	109.5
C10—S4—Eu1	91.30 (16)	H20B—C20—H20C	109.5
C19—S5—Eu1	89.86 (15)	N3—C21—C22	113.9 (4)
C19—S6—Eu1	89.51 (15)	N3—C21—H21A	108.8
C1—N1—C2	122.1 (4)	C22—C21—H21A	108.8
C1—N1—C3	123.1 (4)	N3—C21—H21B	108.8
C2—N1—C3	114.8 (4)	C22—C21—H21B	108.8
C10—N2—C11	121.8 (4)	H21A—C21—H21B	107.7
C10—N2—C12	122.2 (4)	C27—C22—C23	118.3 (5)
C11—N2—C12	115.7 (4)	C27—C22—C21	122.5 (4)
C19—N3—C20	122.8 (4)	C23—C22—C21	119.2 (4)
C19—N3—C21	121.3 (4)	C24—C23—C22	120.4 (6)
C20—N3—C21	115.9 (4)	C24—C23—H23	119.8
C39—N4—C36	118.3 (4)	C22—C23—H23	119.8
C39—N4—Eu1	122.7 (3)	C25—C24—C23	121.0 (6)
C36—N4—Eu1	119.0 (3)	C25—C24—H24	119.5
C28—N5—C32	117.4 (4)	C23—C24—H24	119.5
C28—N5—Eu1	122.1 (3)	C26—C25—C24	118.7 (6)
C32—N5—Eu1	120.3 (3)	C26—C25—H25	120.6
N1—C1—S1	120.1 (3)	C24—C25—H25	120.6
N1—C1—S2	121.0 (3)	C25—C26—C27	121.3 (6)
S1—C1—S2	118.9 (3)	C25—C26—H26	119.3
N1—C2—H2A	109.5	C27—C26—H26	119.3
N1—C2—H2B	109.5	C22—C27—C26	120.2 (5)
H2A—C2—H2B	109.5	C22—C27—H27	119.9
N1—C2—H2C	109.5	C26—C27—H27	119.9
H2A—C2—H2C	109.5	N5—C28—C29	123.3 (5)
H2B—C2—H2C	109.5	N5—C28—H28	118.4
N1—C3—C4	111.7 (4)	C29—C28—H28	118.4
N1—C3—H3A	109.3	C30—C29—C28	119.6 (5)
C4—C3—H3A	109.3	C30—C29—H29	120.2
N1—C3—H3B	109.3	C28—C29—H29	120.2
C4—C3—H3B	109.3	C29—C30—C31	119.6 (4)
H3A—C3—H3B	107.9	C29—C30—H30	120.2
C5—C4—C9	118.2 (5)	C31—C30—H30	120.2
C5—C4—C3	121.1 (4)	C32—C31—C30	117.1 (4)
C9—C4—C3	120.7 (5)	C32—C31—C33	120.2 (5)
C4—C5—C6	120.7 (5)	C30—C31—C33	122.7 (4)
C4—C5—H5	119.7	N5—C32—C31	123.0 (4)
C6—C5—H5	119.7	N5—C32—C36	117.9 (4)
C7—C6—C5	120.4 (6)	C31—C32—C36	119.1 (4)
C7—C6—H6	119.8	C34—C33—C31	121.1 (5)
C5—C6—H6	119.8	C34—C33—H33	119.5
C6—C7—C8	119.5 (6)	C31—C33—H33	119.5
C6—C7—H7	120.3	C33—C34—C35	120.8 (4)
C8—C7—H7	120.3	C33—C34—H34	119.6
C9—C8—C7	120.5 (6)	C35—C34—H34	119.6
C9—C8—H8	119.8	C36—C35—C37	117.7 (4)
C7—C8—H8	119.8	C36—C35—C34	119.7 (4)
C8—C9—C4	120.7 (6)	C37—C35—C34	122.6 (4)
C8—C9—H9	119.6	N4—C36—C35	122.5 (4)
C4—C9—H9	119.6	N4—C36—C32	118.4 (4)
N2—C10—S4	121.0 (4)	C35—C36—C32	119.1 (4)
N2—C10—S3	122.7 (4)	C38—C37—C35	119.7 (4)
S4—C10—S3	116.2 (3)	C38—C37—H37	120.2
N2—C11—H11A	109.5	C35—C37—H37	120.2
N2—C11—H11B	109.5	C37—C38—C39	118.5 (5)
H11A—C11—H11B	109.5	C37—C38—H38	120.8
N2—C11—H11C	109.5	C39—C38—H38	120.8
H11A—C11—H11C	109.5	N4—C39—C38	123.4 (5)
H11B—C11—H11C	109.5	N4—C39—H39	118.3
N2—C12—C13	114.0 (4)	C38—C39—H39	118.3
			
N5—Eu1—S1—C1	−160.64 (18)	C4—C5—C6—C7	0.0 (8)
N4—Eu1—S1—C1	−94.29 (17)	C5—C6—C7—C8	−0.8 (9)
S4—Eu1—S1—C1	103.41 (16)	C6—C7—C8—C9	1.4 (9)
S3—Eu1—S1—C1	113.68 (16)	C7—C8—C9—C4	−1.2 (9)
S2—Eu1—S1—C1	1.20 (16)	C5—C4—C9—C8	0.4 (8)
S5—Eu1—S1—C1	−125.73 (16)	C3—C4—C9—C8	179.1 (5)
S6—Eu1—S1—C1	−28.73 (16)	C11—N2—C10—S4	−3.4 (7)
N5—Eu1—S2—C1	24.9 (2)	C12—N2—C10—S4	170.7 (4)
N4—Eu1—S2—C1	73.14 (18)	C11—N2—C10—S3	−179.8 (4)
S4—Eu1—S2—C1	−67.97 (16)	C12—N2—C10—S3	−5.8 (7)
S3—Eu1—S2—C1	−123.24 (16)	Eu1—S4—C10—N2	−175.9 (4)
S5—Eu1—S2—C1	137.44 (15)	Eu1—S4—C10—S3	0.8 (3)
S6—Eu1—S2—C1	156.03 (16)	Eu1—S3—C10—N2	175.9 (4)
S1—Eu1—S2—C1	−1.19 (15)	Eu1—S3—C10—S4	−0.8 (3)
N5—Eu1—S3—C10	−88.80 (19)	C10—N2—C12—C13	130.3 (5)
N4—Eu1—S3—C10	−139.3 (2)	C11—N2—C12—C13	−55.4 (7)
S4—Eu1—S3—C10	0.48 (17)	N2—C12—C13—C14	115.9 (6)
S2—Eu1—S3—C10	69.71 (18)	N2—C12—C13—C18	−66.7 (7)
S5—Eu1—S3—C10	−158.17 (18)	C18—C13—C14—C15	−0.8 (9)
S6—Eu1—S3—C10	142.26 (17)	C12—C13—C14—C15	176.7 (6)
S1—Eu1—S3—C10	−10.51 (19)	C13—C14—C15—C16	1.3 (9)
N5—Eu1—S4—C10	93.77 (18)	C14—C15—C16—C17	0.2 (11)
N4—Eu1—S4—C10	142.8 (2)	C15—C16—C17—C18	−2.0 (12)
S3—Eu1—S4—C10	−0.48 (17)	C14—C13—C18—C17	−1.1 (10)
S2—Eu1—S4—C10	−130.01 (17)	C12—C13—C18—C17	−178.5 (6)
S5—Eu1—S4—C10	26.39 (18)	C16—C17—C18—C13	2.5 (12)
S6—Eu1—S4—C10	−60.29 (17)	C20—N3—C19—S5	177.6 (4)
S1—Eu1—S4—C10	170.70 (17)	C21—N3—C19—S5	−0.7 (6)
N5—Eu1—S5—C19	152.45 (16)	C20—N3—C19—S6	−2.1 (6)
N4—Eu1—S5—C19	84.61 (16)	C21—N3—C19—S6	179.6 (3)
S4—Eu1—S5—C19	−131.30 (14)	Eu1—S5—C19—N3	−170.0 (3)
S3—Eu1—S5—C19	−106.71 (14)	Eu1—S5—C19—S6	9.6 (2)
S2—Eu1—S5—C19	14.80 (15)	Eu1—S6—C19—N3	170.0 (3)
S6—Eu1—S5—C19	−5.74 (14)	Eu1—S6—C19—S5	−9.6 (2)
S1—Eu1—S5—C19	116.31 (14)	C19—N3—C21—C22	95.4 (5)
N5—Eu1—S6—C19	−21.16 (18)	C20—N3—C21—C22	−83.0 (5)
N4—Eu1—S6—C19	−69.62 (16)	N3—C21—C22—C27	−31.9 (7)
S4—Eu1—S6—C19	124.76 (14)	N3—C21—C22—C23	150.1 (5)
S3—Eu1—S6—C19	75.32 (14)	C27—C22—C23—C24	−0.7 (9)
S2—Eu1—S6—C19	−159.18 (14)	C21—C22—C23—C24	177.3 (6)
S5—Eu1—S6—C19	5.73 (14)	C22—C23—C24—C25	0.2 (11)
S1—Eu1—S6—C19	−132.17 (14)	C23—C24—C25—C26	0.3 (11)
N5—Eu1—N4—C39	−178.8 (4)	C24—C25—C26—C27	−0.3 (11)
S4—Eu1—N4—C39	124.3 (3)	C23—C22—C27—C26	0.8 (9)
S3—Eu1—N4—C39	−120.3 (3)	C21—C22—C27—C26	−177.3 (5)
S2—Eu1—N4—C39	36.1 (4)	C25—C26—C27—C22	−0.3 (10)
S5—Eu1—N4—C39	−101.5 (4)	C32—N5—C28—C29	−2.2 (7)
S6—Eu1—N4—C39	−39.2 (4)	Eu1—N5—C28—C29	−177.5 (4)
S1—Eu1—N4—C39	97.3 (4)	N5—C28—C29—C30	1.5 (8)
N5—Eu1—N4—C36	−1.9 (3)	C28—C29—C30—C31	−0.9 (8)
S4—Eu1—N4—C36	−58.7 (4)	C29—C30—C31—C32	1.1 (7)
S3—Eu1—N4—C36	56.6 (4)	C29—C30—C31—C33	179.0 (5)
S2—Eu1—N4—C36	−147.0 (3)	C28—N5—C32—C31	2.4 (6)
S5—Eu1—N4—C36	75.4 (3)	Eu1—N5—C32—C31	177.8 (3)
S6—Eu1—N4—C36	137.8 (3)	C28—N5—C32—C36	−176.8 (4)
S1—Eu1—N4—C36	−85.8 (3)	Eu1—N5—C32—C36	−1.5 (5)
N4—Eu1—N5—C28	176.8 (4)	C30—C31—C32—N5	−1.9 (6)
S4—Eu1—N5—C28	−34.9 (3)	C33—C31—C32—N5	−179.9 (4)
S3—Eu1—N5—C28	26.6 (4)	C30—C31—C32—C36	177.4 (4)
S2—Eu1—N5—C28	−127.5 (3)	C33—C31—C32—C36	−0.7 (6)
S5—Eu1—N5—C28	96.7 (4)	C32—C31—C33—C34	1.4 (7)
S6—Eu1—N5—C28	121.5 (3)	C30—C31—C33—C34	−176.6 (5)
S1—Eu1—N5—C28	−104.2 (3)	C31—C33—C34—C35	−0.5 (8)
N4—Eu1—N5—C32	1.7 (3)	C33—C34—C35—C36	−1.0 (7)
S4—Eu1—N5—C32	150.0 (3)	C33—C34—C35—C37	−180.0 (5)
S3—Eu1—N5—C32	−148.5 (3)	C39—N4—C36—C35	0.3 (6)
S2—Eu1—N5—C32	57.4 (3)	Eu1—N4—C36—C35	−176.8 (3)
S5—Eu1—N5—C32	−78.4 (3)	C39—N4—C36—C32	179.1 (4)
S6—Eu1—N5—C32	−53.7 (3)	Eu1—N4—C36—C32	2.0 (5)
S1—Eu1—N5—C32	80.7 (3)	C37—C35—C36—N4	−0.5 (6)
C2—N1—C1—S1	2.4 (6)	C34—C35—C36—N4	−179.5 (4)
C3—N1—C1—S1	−178.1 (4)	C37—C35—C36—C32	−179.3 (4)
C2—N1—C1—S2	−176.7 (4)	C34—C35—C36—C32	1.7 (6)
C3—N1—C1—S2	2.8 (6)	N5—C32—C36—N4	−0.4 (6)
Eu1—S1—C1—N1	178.8 (4)	C31—C32—C36—N4	−179.7 (4)
Eu1—S1—C1—S2	−2.0 (3)	N5—C32—C36—C35	178.5 (4)
Eu1—S2—C1—N1	−178.8 (4)	C31—C32—C36—C35	−0.8 (6)
Eu1—S2—C1—S1	2.0 (3)	C36—C35—C37—C38	0.4 (7)
C1—N1—C3—C4	−113.7 (5)	C34—C35—C37—C38	179.4 (5)
C2—N1—C3—C4	65.9 (6)	C35—C37—C38—C39	0.0 (8)
N1—C3—C4—C5	64.9 (6)	C36—N4—C39—C38	0.1 (7)
N1—C3—C4—C9	−113.8 (5)	Eu1—N4—C39—C38	177.1 (4)
C9—C4—C5—C6	0.2 (8)	C37—C38—C39—N4	−0.3 (8)
C3—C4—C5—C6	−178.4 (5)		
